# Influence of Local Burning on Difference Reflectance Indices Based on 400–700 nm Wavelengths in Leaves of Pea Seedlings

**DOI:** 10.3390/plants10050878

**Published:** 2021-04-27

**Authors:** Ekaterina Sukhova, Lyubov Yudina, Ekaterina Gromova, Anastasiia Ryabkova, Vladimir Vodeneev, Vladimir Sukhov

**Affiliations:** Department of Biophysics, N.I. Lobachevsky State University of Nizhny Novgorod, 603950 Nizhny Novgorod, Russia; lyubovsurova@mail.ru (L.Y.); kater333@inbox.ru (E.G.); nastay2903@bk.ru (A.R.); v.vodeneev@mail.ru (V.V.); vssuh@mail.ru (V.S.)

**Keywords:** variation potential, reflectance indices, photosynthetic response, remote sensing, pea leaves

## Abstract

Local damage (e.g., burning) induces a variation potential (VP), which is an important electrical signal in higher plants. A VP propagates into undamaged parts of the plant and influences numerous physiological processes, including photosynthesis. Rapidly increasing plant tolerance to stressors is likely to be a result of the physiological changes. Thus, developing methods of revealing VP-induced physiological changes can be used for the remote sensing of plant systemic responses to local damage. Previously, we showed that burning-induced VP influenced a photochemical reflectance index in pea leaves, but the influence of the electrical signals on other reflectance indices was not investigated. In this study, we performed a complex analysis of the influence of VP induction by local burning on difference reflectance indices based on 400–700 nm wavelengths in leaves of pea seedlings. Heat maps of the significance of local burning-induced changes in the reflectance indices and their correlations with photosynthetic parameters were constructed. Large spectral regions with significant changes in these indices after VP induction were revealed. Most changes were strongly correlated to photosynthetic parameters. Some indices, which can be potentially effective for revealing local burning-induced photosynthetic changes, are separately shown. Our results show that difference reflectance indices based on 400–700 nm wavelengths can potentially be used for the remote sensing of plant systemic responses induced by local damages and subsequent propagation of VPs.

## 1. Introduction

Local actions of stressors on plants require systemic adaptive responses based on the generation and propagation of long-distance stress signals. Electrical signals (ESs), including action potential, system potential, and variation potential (VP) [1,2,3,4,5,6,7,8,9], play an important role in the induction of physiological changes in non-irritated parts of plants. It is known that an action potential is a self-propagating depolarization electrical signal [1,2,3,6,10] induced by non-damaging stimuli and is caused by both transient activation of Ca^2+^, K^+^, and anion channels, and inactivation of H^+^-ATPase in the plasma membrane. The system potential is a weakly investigated hyperpolarization signal [8,11,12] caused by transient activation of H^+^-ATPase and, possibly, changes in activity K^+^ channels.

A VP is a long-distance signal in higher plants induced by local damage [2,4,6,13], which is formed by long-term depolarization and short-term “AP-like” spikes. The generation of a VP is mainly based on transient inactivation of H^+^-ATPase, induced by Ca^2+^ influx through Ca^2^ channels [2,13], but anion and outward K^+^ channels can participate in the generation of AP-like spikes [4]. The mechanisms of VP propagation are still being discussed. The first hypothesis [13,14,15,16] supposes that a VP is a local electrical response induced by a hydraulic wave, which propagates through a plant from the damaged zone and activates mechanosensitive Ca^2+^ channels. The second hypothesis [4,17] supposes that the VP is the local electrical response induced by the propagation of a specific “wound substance” from the damaged zone and the activation of ligand-dependent Ca^2+^ channels. Additionally, there are hypotheses of VP propagation based on combinations of hydraulic and chemical signals [18,19,20,21].

ESs induced by local stimuli can strongly influence physiological processes in non-irritated parts of plants [2,3,5,7,8]. It is known that ESs increase the expression of defense genes [22,23,24,25], respiration [26,27,28], ATP content in leaves [28], production of phytohormones [9,25,29,30,31,32], etc. In contrast, other physiological processes (e.g., phloem loading [33], phloem mass flow [34,35], and plant growth [36]) can be suppressed after propagation of ESs. Photosynthesis is an important target of ESs [5]. It is known that ESs decrease photosynthetic CO_2_ assimilation, quantum yield of photosystem I, quantum yield of photosystem II (Y(II)), and mesophyll CO_2_ conductance [23,27,37,38,39,40,41,42,43]. Non-photochemical quenching of chlorophyll fluorescence (NPQ), cyclic electron flow, and photosynthetic light absorption can be stimulated by ESs [27,37,39,43,44,45]. Increasing plant tolerance to stressors (including tolerance of photosynthetic machinery) is likely the result of physiological changes [46,47,48,49,50,51,52,53].

It can be expected that monitoring of electrical activity and ES-induced physiological changes is a potential tool for revealing the actions of stressors on plants. Investigations of plant electrical activity show that (i) the total electrical activity of plants (“electrome”) can be strongly dependent on abiotic and biotic factors [53,54,55,56,57], and (ii) analysis of the electrical activity can be used for the classification of stressors that act on plants [58,59,60,61,62]. However, direct measurements of electrical activity cannot be used for the remote sensing of ES-induced systemic responses because electrodes would need to be connected to the plant. An alternative approach can be based on revealing relationships between ES-induced physiological changes and changes in plant reflectance. Previously, we showed that ESs induce changes in broadband reflectance indices [63,64] and decrease some narrowband indices, including the water index [64], which shows the water content in leaves, and the photochemical reflectance index (PRI) [65]. The PRI is strongly related to photosynthetic processes [66,67,68,69,70,71,72,73,74], including Y(II) and NPQ [75,76,77]. Its fast changes (seconds and minutes) are caused by the acidification of a chloroplast lumen through transitions in the xanthophyll cycle [66,68,78] and a change in light scattering at 530–546 nm [68,77], which is likely to be caused by chloroplast shrinkage [75,77]. It is known that ESs are accompanied by pH decreases in the cytoplasm, stroma, and lumen [38,39,79]; the luminal pH decrease is likely to be a mechanism of the ES’s influence on the PRI.

However, using only the current narrowband reflectance indices (RIs) can limit the search for new RIs that are sensitive to the ES-induced physiological changes, because the reflected light at most wavelengths is not analyzed in standard RIs. The limitation can be eliminated by using heat maps showing (i) the correlation coefficients of physiological parameters to all possible RIs calculated on the basis of measured spectra [80,81,82] or (ii) the significance of changes in these RIs under the action of stressors [83]. Considering these approaches, it can be expected that a similar analysis of RIs in the 400–700 nm spectral range, which is related to photosynthetic processes, could reveal new RIs that are sensitive to ES-induced photosynthetic changes.

The aim of the current work was to perform a complex analysis of the influence of local burning, which is a typical inductor of VP (a key ES in higher plants [5,8]), on difference reflectance indices based on 400–700 nm wavelengths in leaves of pea seedlings. The analysis was based on spectra and photosynthetic parameters, which were preliminarily measured in our work [65] devoted to VP influence on the PRI. The spectra were used for the calculation of all probable RIs, and their changes after VP induction and correlations with photosynthetic parameters were investigated.

## 2. Data Analysis

### 2.1. General Scheme of Data Analysis

We used spectral and photosynthetic data from [65] in our analysis. Only data taken from the second leaf were analyzed, because the local burning-induced electrical signals and photosynthetic and PRI changes in the fourth leaf [65] were weak. All spectra of reflected light and photosynthetic parameters measured in the second leaves were analyzed as a single experimental group (i.e., similar experimental groups from [65] were combined into one group). This group included 13 repetitions.

**Figure 1:**
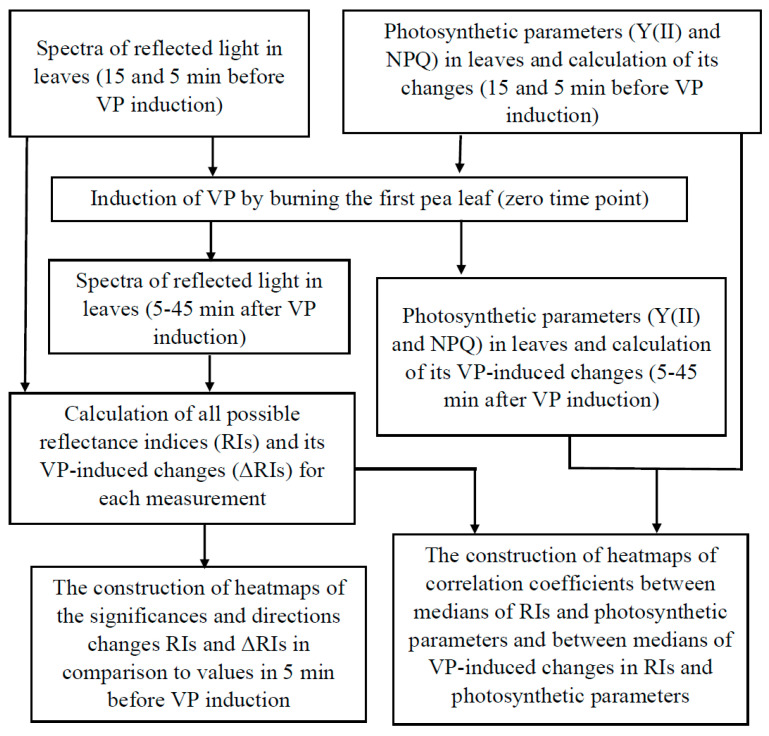
General scheme of analysis of spectra of the reflected light and photosynthetic parameters (the quantum yield of photosystem II, Y(II), and non-photochemical quenching of chlorophyll fluorescence, NPQ) measured in the second pea leaf before and after induction of variation potential (VP) by local burning of the first leaf.

Figure 1 shows the scheme of the data analysis. Seven time points were used, including two points before VP induction (15 and 5 min before) and five points after the induction (5, 15, 25, 35, and 45 min after). We previously showed that RIs calculated on the basis of 400–700 nm wavelengths can have non-normal distributions [83]. Thus, we used non-parametric statistics in our analysis. Medians of the investigated values were used as the non-parametric analog of averaged values.

The values of the investigated parameters 5 min before VP induction were used as control values for each plant. We calculated the absolute values of the investigated parameters (RIs, Y(II), and NPQ) and their changes (differences between current and control values of the parameters in each plant, ΔRIs, ΔY(II), and ΔNPQ). Significant differences between values were calculated using the non-parametric Mann–Whitney U test. Relationships between reflectance indices and photosynthetic parameters were estimated on the basis of Pearson’s correlation coefficients. Medians, which were separately calculated on the basis of the absolute values of the parameters or their changes for each time point, were used for the calculation (*n* = 7).

### 2.2. Calculation of Difference Reflectance Indices and Construction of Heat Maps

The calculation of RIs (or ΔRIs) and the construction of heat maps were based on several programs, which were developed using the Python 3.8 programming language. They solved the following tasks:

(i) Calculation of all possible RIs on the basis of Equation (1):

(1) $$\mathrm{RI}=\frac{I\left(R_{1}\right)-I\left(R_{2}\right)\frac{I_{C}{(R}_{1})}{I_{C}{(R}_{2})}}{I\left(R_{1}\right)+I\left(R_{2}\right)\frac{I_{C}{(R}_{1})}{I_{C}{(R}_{2})}},$$

where I(R_1_) and I(R_2_) are the intensities of the reflected light from a leaf at R_1_ and R_2_ wavelengths, respectively; I_C_(R_1_) and I_C_(R_2_) are the intensities of the reflected light from a white reflectance standard at R_1_ and R_2_ wavelengths (in accordance with [76,84]), respectively. To increase accuracy, averaged intensities of reflected light (3 nm range) were used. RIs were not calculated at R_2_ ≥ R_1_. Changes in RIs (ΔRIs) were calculated according to Equation (2):

(2) $$\Delta \mathrm{RI}={\mathrm{RI}}_{\mathrm{TP}}-{\mathrm{RI}}_{C},$$

where RI_TP_ is the RI at a specific time point, and RI_C_ is the control RI equal to the RI at 5 min before VP induction.

(ii) Calculation of the significance (*p*) of differences between experimental and control values of RIs (or ΔRIs) on the basis of the non-parametric Mann–Whitney U test and estimation of the directions of the differences. Two-dimensional data arrays (significance and directions of changes for each RI as a function of R_1_ and R_2_) were used for the construction of heat maps.

(iii) Calculation of the medians of RIs (or ΔRIs) at each time point and Pearson’s correlation coefficients of these medians to similar medians of Y(II) and NPQ. Two-dimensional data arrays (correlation coefficients for each RI as a function of R_1_ and R_2_) were used for the construction of heat maps.

## 3. Results

### 3.1. Local Burning-Induced Changes in Difference Reflectance Indices

**Figure 2:**
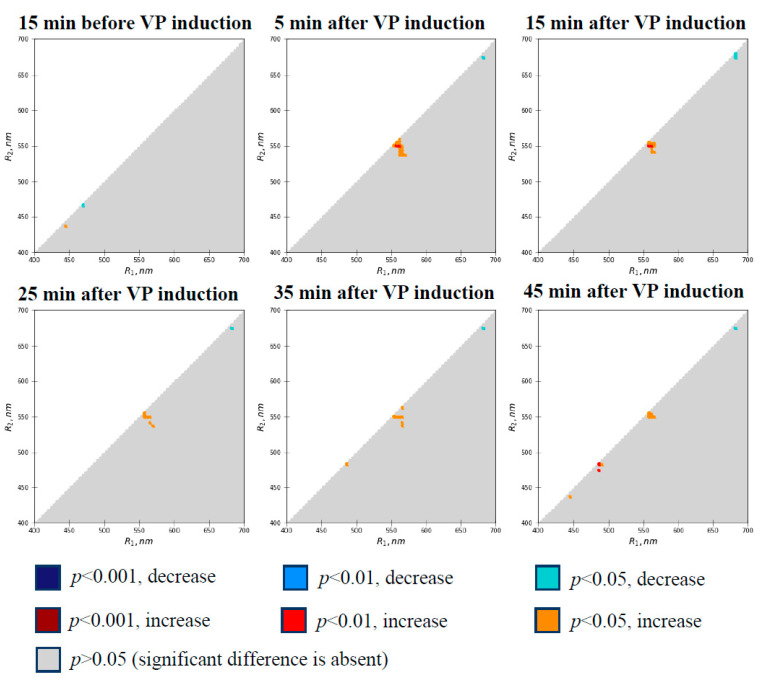
Heat maps of significance and directions of changes in absolute values of RIs in the second pea leaf at different time intervals before and after induction of variation potential (*n* = 13). RIs were calculated based on Equation (1). Burning of the first leaf was used for VP induction. The Mann–Whitney U test was used for *p*-value calculations. The absolute values of RIs were compared to the values of RIs at 5 min before VP induction.

Figure 2 shows the heat maps of the significance and directions of differences between the absolute values of RIs at different time points and the control values. It is shown that differences were absent in the RIs before VP induction. Induction of a VP by local burning caused a transient increase in RIs (mostly at 5 and 15 min after heating) in a small spectral range (R_1_ was about 550–570 nm; R_2_ was about 535–560 nm). The increase in RIs approximately corresponded to the decrease in the PRI, which was shown in [65]. The opposite direction of RI changes is related to the opposite order of R_1_ and R_2_, because the PRI based on R_1_ = 531 nm and R_2_ = 570 nm [65] corresponds to the RI based on R_1_ = 570 nm and R_2_ = 531 nm in Figure 2. It is interesting that there were extremely small areas (pixel level) showing significant changes in RIs in other spectral ranges (e.g., RIs based on R_1_ equal to about 680 nm and R_2_ equal to about 675 nm; the measured reflected light at the wavelengths can additionally include chlorophyll fluorescence).

**Figure 3:**
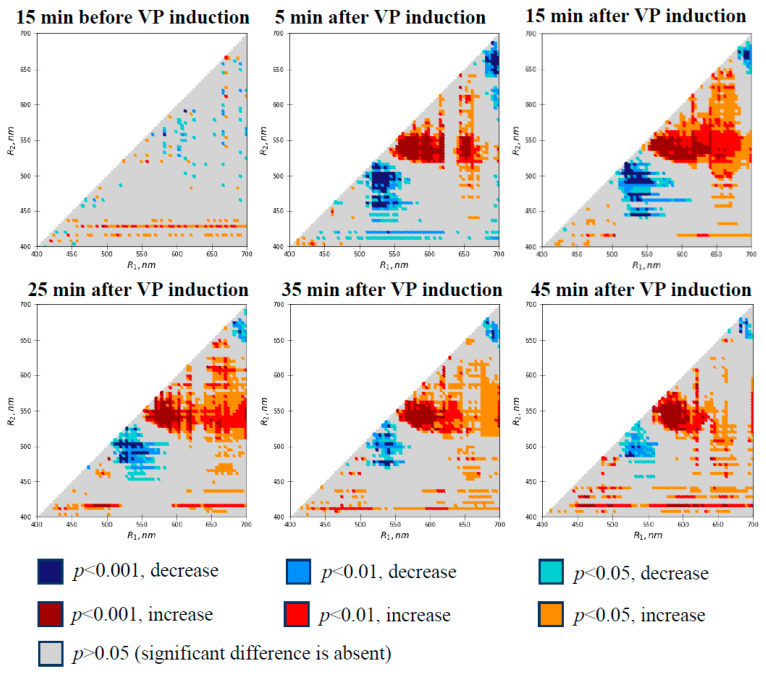
Heat maps of significance and directions of changes in ΔRIs in the second pea leaf at different time intervals before and after induction of variation potential (*n* = 13). RIs were calculated based on Equation (1). Burning of the first leaf was used for VP induction. Each ΔRI was calculated as RI_TP_–RI_C_. RI_C_ is the control RI at 5 min before the VP induction. RI_TP_ is the difference in the reflectance index at a specific time point before or after VP induction. The Mann–Whitney U test was used for *p*-value calculations. The ΔRIs were compared to zero values (absence of changes). Using ΔRIs minimized the influence of individual plant differences on the local burning-induced changes in RIs.

Previously, we showed that changes in RIs (e.g., PRI or broadband reflectance indices) were more sensitive to short-term actions of stressors [63,64,65,76,83,85] than their absolute values, because using ΔRIs excluded the individual variability of the initial values of RIs and decreased the standard errors of the measured values. The effect was also observed in works by other authors (e.g., [74,86] for PRI). Thus, we analyzed the significance of the changes in ΔRIs in a further analysis and constructed heat maps of the significance of changes in ΔRIs (Figure 3).

There were only separate pixels on the first heat map showing a significant difference in ΔRIs at 15 min before VP induction (Figure 3). The localizations of the pixels were rather chaotic, excluding a few pixel lines in the lower part of the figure. It is also important to note that RIs with highly significant differences (*p* < 0.001) were practically absent in the variants (about 0.1% of the total quantity of RIs). Considering these results, we supposed that the changes in RIs were mainly related to revealing false changes, which were caused by stochastic differences in the spectra measured at different time intervals and the large quantity of simultaneously analyzed RIs. The false changes could be the result of cooperative effects of moderate stochastic differences in light measurements at both wavelengths (separate pixels) or high stochastic differences at a single wavelength (line of pixels).

VP induction by local burning strongly influenced ΔRIs (Figure 3). There were several large spectral regions in the heat maps with significant positive changes in ΔRIs (e.g., R_1_ was about 540–625 nm and R_2_ was about 520–560 nm), in addition to negative changes (e.g., R_1_ was about 510–560 nm and R_2_ was about 450–520 nm). The area of the spectral regions was dependent on the duration after VP induction. For example, ΔRIs with highly significant changes (*p* < 0.001) accounted for 8–9% of the total quantity of ΔRIs at 5 and 15 min after burning and for approximately 4–5% at 35 and 45 min.

The results show that the induction of VP by local burning caused changes in a large number of ΔRIs and weakly influenced the absolute values of RIs. The changes could be related to local burning-induced photosynthetic changes in pea leaves; as a result, an analysis of the relations of RIs and ΔRIs to photosynthetic parameters was the next task of investigation.

### 3.2. Relations of Local Burning-Induced Changes in Difference Reflectance Indices to Changes in Photosynthetic Parameters

**Figure 4:**
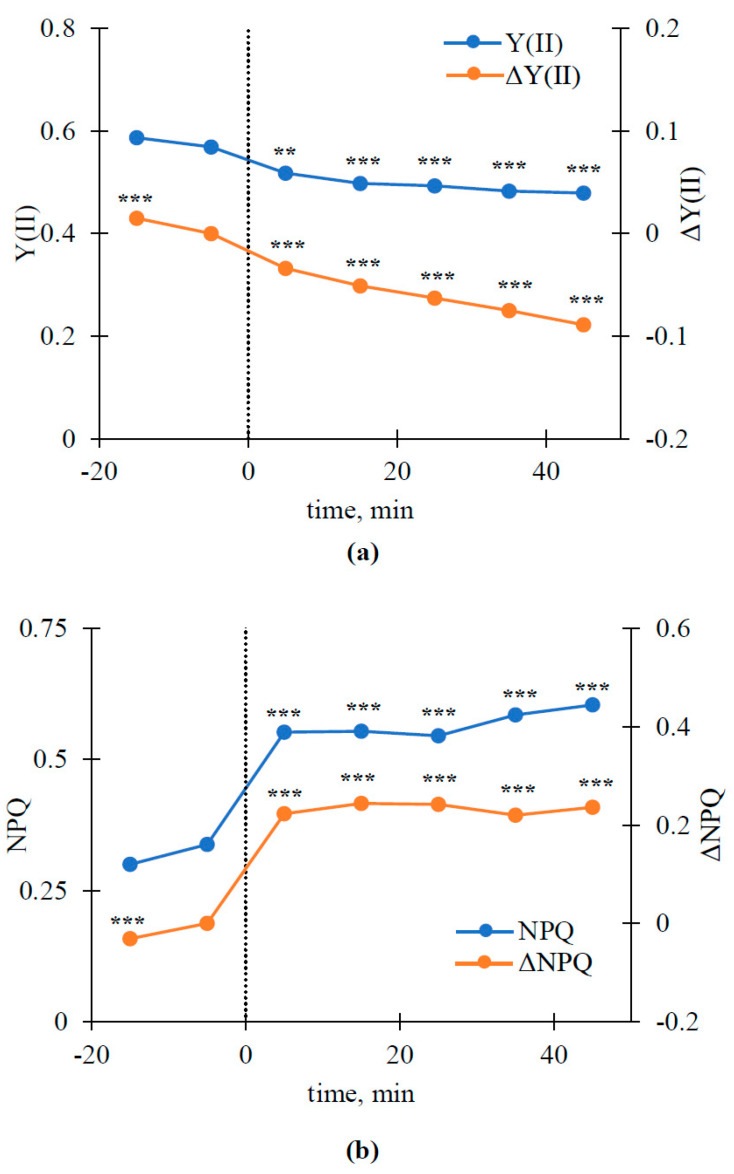
Dynamics of Y(II) and ΔY(II) (**a**) and NPQ and ΔNPQ (**b**) before and after induction of variation potential in the second leaf (*n* = 13). Burning the first leaf was used for VP induction (the burning is marked by the time point zero and dotted line). ΔY(II) and ΔNPQ were calculated as Y(II)_TP_-Y(II)_C_ and NPQ_TP_-NPQ_C_, respectively. Y(II)_C_ is the control Y(II) at 5 min before VP induction. Y(II)_TP_ is Y(II) at a specific time point before or after VP induction. NPQ_C_ is the control NPQ at 5 min before VP induction. NPQ_TP_ is NPQ at a specific time point before or after VP induction. The Mann–Whitney U test was used for *p*-value calculations. Y(II) and NPQ were compared to control values. ΔY(II) and ΔNPQ were compared to zero values (absence of changes). Using ΔY(II) and ΔNPQ minimized the influence of individual plant differences on local burning-induced changes in the parameters. **, *p* < 0.01 and ***, *p* < 0.001.

Figure 4 shows the absolute values of Y(II) and NPQ and changes in the parameters (ΔY(II) and ΔNPQ) before and after the induction of variation potential by burning the leaflet of the first pea leaf. The photosynthetic parameters were calculated on the basis of all photosynthetic responses in the second pea leaf, which were shown in previous work [65].

It was shown that the VP induction by local burning caused a fast decrease in the absolute value of Y(II) (Figure 4a) and increased the absolute value of NPQ (Figure 4b); both changes were significant. The result was in a good agreement with numerous works (e.g., see the review in [5]) devoted to investigating the influence of ESs on photosynthetic processes. The analysis of ΔY(II) and ΔNPQ showed similar changes in the parameters after the VP induction. However, small significant changes in ΔY(II) (decrease) and ΔNPQ (increase) were also observed before the induction of the variation potential. The last result showed slow changes in the investigated photosynthetic parameters (especially, ΔY(II)) before burning, which was in accordance with several works (e.g., [39,40,48,49,87]). The changes were related to the slow photosynthetic response (tens of minutes) induced by illumination with high intensity (probably by the photosynthetic state transition and (or) photodamage).

**Figure 5:**
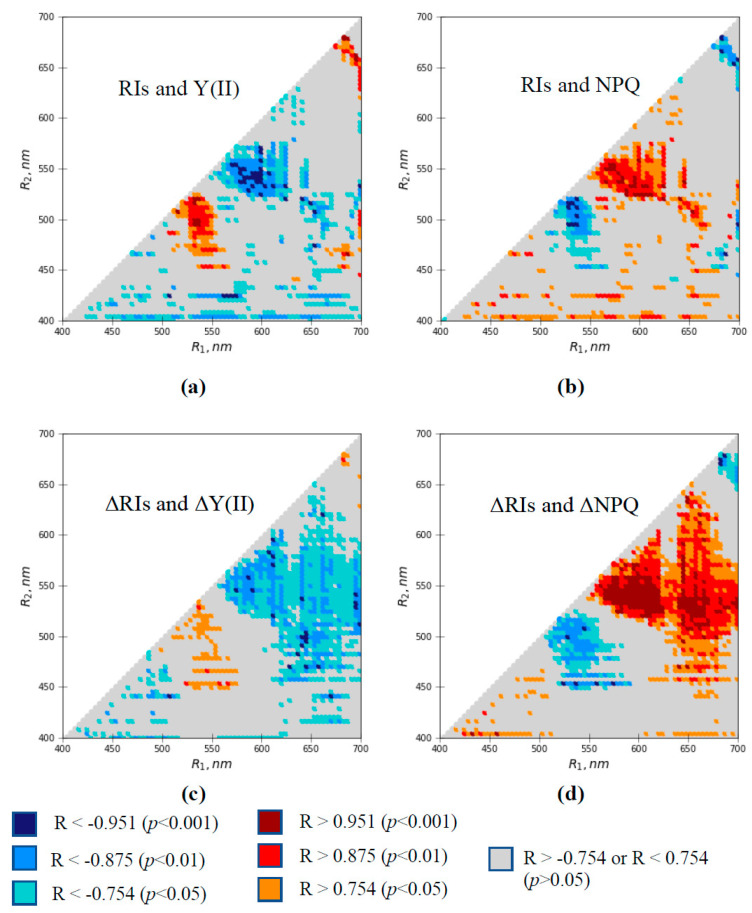
Heat maps of linear correlation coefficients (R) between RIs and Y(II) (**a**), RIs and NPQ (**b**), ΔRIs and ΔY(II) (**c**), and ΔRIs and ΔY(II) (**d**). Pearson’s correlation coefficients were calculated based on medians of investigated parameters. The medians were calculated for each time point (*n* = 7). Only significant correlation coefficients are shown in the figure.

Figure 5 shows heat maps of Pearson’s correlation coefficients of RIs with Y(II) and NPQ and the correlation of ΔRIs with ΔY(II) and ΔNPQ. Only significant correlation coefficients are shown in the figure.

It was shown that both RIs and ΔRIs were strongly correlated to photosynthetic parameters in large spectral regions. In particular, modules of correlation coefficients could be more than 0.95 in several spectral regions (e.g., R_1_ is about 595–620 nm and R_2_ is about 525–540 nm in Figure 5d). The relations of RIs and ΔRIs to photosynthetic parameters could be positive and negative in different spectral regions. Relationships of reflectance indices to the quantum yield of photosystem II and non-photochemical quenching were mainly opposite, which is in good agreement with the opposing direction of the changes in Y(II) and NPQ (Figure 4).

Areas of the spectral regions with a significant correlation of ΔRIs with ΔY(II) and ΔNPQ were larger than the areas with a significant correlation of RIs with Y(II) and NPQ. The results support the conclusion that the sensitivity of ΔRIs to photosynthetic parameters is higher than the sensitivity of RIs.

Spectral regions with a significant correlation of RIs and ΔRIs with photosynthetic parameters (e.g., R_1_ was about 550–620 nm and R_2_ was about 500–575 nm, or R_1_ was about 510–560 nm and R_2_ was about 460–520 nm; Figure 5d) were similar to the spectral regions with significant changes in ΔRIs after the induction of VP (Figure 3). This showed that photosynthetic changes were likely to be the causes of the revealed changes in RIs, which were observed after the induction of the VP in pea leaves.

### 3.3. Dynamics of Local Burning-Induced Changes in Some Revealed Reflectance Indices

**Figure 6:**
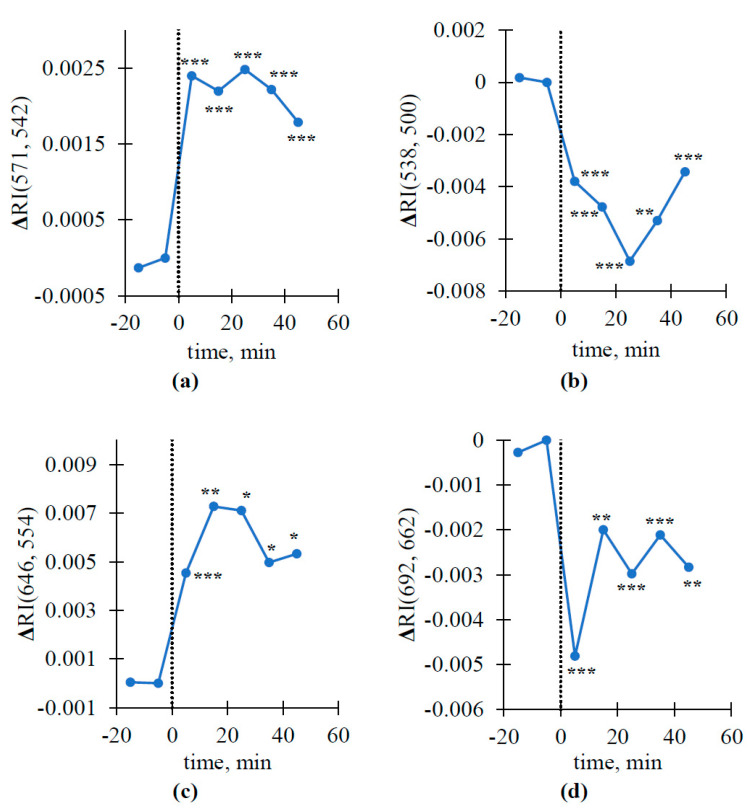
Dynamics of ΔRI(571, 542) (**a**), ΔRI(538, 500) (**b**), ΔRI(646, 554) (**c**), and ΔRI(692, 662) (**d**) before and after induction of variation potential in the second leaf (*n* = 13). Burning of the first leaf was used for VP induction (the burning is marked by the time point zero and the dotted line). The Mann–Whitney U test was used for *p*-value calculations; parameters were compared to zero values (absence of changes). *, *p* < 0.05, **, *p* < 0.01, and ***, *p* < 0.001.

Furthermore, we analyzed the dynamics of local burning-induced changes in some reflectance indices, which were included in spectral areas with significant changes (Figure 6). Only ΔRIs were analyzed because changes in the absolute values of RIs were weak. The dynamics of burning-induced changes in ΔRI(571, 542) (R_1_ was 571 nm and R_2_ was 542 nm), ΔRI(538, 500) (R_1_ was 538 nm and R_2_ was 500 nm), ΔRI(646, 554) (R_1_ was 646 nm and R_2_ was 554 nm), and ΔRI(692, 662) (R_1_ was 692 nm and R_2_ was 662 nm), which were selected on the basis of different spectral ranges in Figure 3, were investigated.

It was shown that all investigated ΔRIs were strongly changed after VP induction. The magnitudes of the changes varied from approximately 0.0025 for ΔRI(571, 542) to 0.007–0.008 for ΔRI(538, 505) and ΔRI(646, 554), similar to the magnitudes of VP-induced changes in PRI [65]. The dynamics of the changes in different ΔRIs also differed: the minimum values of ΔRI(538, 500) and ΔRI(692, 662) were observed at 25 and 5 min after VP induction, respectively. The maximum value of ΔRI(646, 554) was observed at 15 min after burning. ΔRI(571, 542) reached maximal values at 5 min after the induction of variation potential, which were weakly changed after that.

## 4. Discussion

Damage-induced VP is a key electrical signal in higher plants influencing numerous physiological processes and, probably, participating in the induction of the systemic adaptive response [2,5,7,8]. Photosynthetic processes are an important target of VP [2,3,5,7,8,27,28,30,31,37,38,39,40,41,42,43,44,45,48]. Photosynthetic inactivation can be initiated for minutes after VP induction; both dark and light reactions are changed, modifications in photosynthetic processes participate in increasing tolerance of photosynthetic machinery, etc. The VP’s influence on photosynthetic processes is based on the pH increase in the apoplast and the pH decrease in the cytoplasm, stroma, and lumen [5,8,38,39,79].

It is known that photosynthetic processes are strongly related to plant optical properties, including fast changes in leaf reflectance (e.g., reflectance in the green spectral range [66,68,75,76,77,78,88,89,90], which is caused by acidification of the lumen of chloroplasts). Thus, it seems highly probable that VP-induced photosynthetic changes should be accompanied by changes in leaf reflectance, which can potentially be used for their remote sensing.

Our previous results showed that the local burning-induced VP causes changes in broadband reflectance indices and the narrowband water index [63,64], which are related to the water content in leaves, and decrease in the narrowband PRI [65], which is correlated to the quantum yield of photosystems and non-photochemical quenching. Potentially, the VP can induce changes in other difference reflectance indices because the changes are observed under direct actions of stressors (e.g., heating [83]); however, the problem requires an additional complex analysis, such as the analyses in works [80,81,82,83]. In the present work, we performed an analysis based on spectral and photosynthetic data, which were obtained from our previous work [65]. We calculated all possible RIs and ΔRIs based on the 400–700 nm spectral range, revealed their changes caused by local burning (the typical inductor of VP [4]), and estimated the relation of the RIs and ΔRIs to the quantum yield of photosystem II and non-photochemical quenching.

The complex analysis shows that the induction of VP by local burning causes significant changes in a large quantity of ΔRIs (Figure 3); in contrast, the changes in RIs were weak (Figure 2). This is in good agreement with results showing that fast changes in PRI (e.g., light-induced [74,76,77,85,86] or VP-induced [65] changes) are more effective estimators of photosynthetic parameters than absolute values of the index. The effect is based on the elimination of individual variability in reflectance spectra related to long-term changes in physiological processes (e.g., content of chlorophylls and carotenoids [71,74]). It is highly likely that a similar mechanism could also decrease errors in our analysis, which is supported by the increased sensitivity of ΔRIs in comparison to the absolute values of RIs in the complex analysis of the spectra of reflected light after short-term heating of plants [83].

Many spectral regions with significant changes in ΔRIs are based in the green and yellow spectral ranges (at least one of two R values is in the 500–600 nm range; Figure 3 and Figure 6); high Pearson’s correlation coefficients were also observed in this spectral range (Figure 5). The changes in reflectance indices can be explained by transitions in the xanthophyll cycle because they are sensitive to the photosynthetic decrease in pH in the lumen [88,91,92] and modify reflectance in the 510–560 nm range [66,78]. However, the maximum reflectance was observed at 525–535 nm [66,68,78]. This means that additional mechanisms of change in reflectance in the green and yellow spectral ranges are probable.

Potentially, the mechanisms could be related to light scattering, with maximum values at 530–546 nm, which is dependent on the pH in the lumen (probably through the induction of chloroplast shrinkage [77]) and can influence reflectance [68,75,77,79]. Previously, we showed [79] that light scattering can be stimulated by a VP in peas, and the dynamics of VP-induced changes in light scattering (see, e.g., Figure 7a in [79]) seem to be approximately similar to the dynamics of changes in ΔRI (646, 554) (Figure 6c). It is also known that light scattering is strongly related to NPQ [93,94]; i.e., it should show fast changes in photosynthetic processes.

Another potential mechanism of changes in reflectance can be related to an electrochromic shift in pigment absorbance with maximum values at 515–520 nm [94,95]. The electrochromic shift is caused by electrical potential across thylakoid membranes in chloroplasts [94,95]; this means that this parameter can be also related to photosynthesis. Our earlier results [79] showed that a VP decreases the electrochromic shift in pea leaves, but the magnitude of the decrease is small.

All potential mechanisms (transitions in the xanthophyll cycle, light scattering, and the electrochromic shift) induce changes in ΔRIs as a result of photosynthetic processes, including lumen acidification and formation of electrical potential across thylakoid membranes [92,93,94,95]. The correlations between photosynthetic parameters and changes in ΔRIs (Figure 5) support this mechanism.

However, changes in RIs (Figure 3 and Figure 6d) and correlations (Figure 5) were also observed in red spectral regions (R_1_ was about 680–700 nm and R_2_ was about 645–675 nm). It should be noted that we cannot divide reflected light itself and fluorescence in the measured reflected light. The maximum photosystem II fluorescence is known to be observed at 685 nm, and chlorophyll absorbance is shown in the blue and red spectral ranges [88]. As a result, we hypothesize that negative changes in ΔRIs based in the red spectral region (see, e.g., Figure 3) show a VP-induced decrease in chlorophyll fluorescence, which is included in I(R_1_), in comparison to I(R_2_), which is mainly dependent on chlorophyll light absorption. The hypothesis is in good agreement with works [27,37,39,43,44,45] showing that the non-photochemical quenching of chlorophyll fluorescence can be strongly stimulated by electrical signals [27,37,39,43,44,45]. The strong negative correlation between RIs (or ΔRIs) and NPQ (or ΔNPQ) in the red spectral range also supports the hypothesis about the participation of the decrease in fluorescence intensity in the VP-induced decrease in RIs based in this spectral region. It should be noted that the VP-induced NPQ increase is also caused by acidification of the chloroplast lumen. The mechanism can contribute to relations between RIs based in the red and green–yellow spectral regions.

Thus, our analysis shows that local burning-induced VP causes changes in a large quantity of ΔRIs, which are mainly related to changes in reflectance in the green and yellow spectral ranges; however, ΔRIs can also be significantly changed in the red spectral range. In the future, these results can provide the basis for the development of new indices for the remote sensing of ES-induced photosynthetic changes in plant leaves. In contrast, absolute values of RIs are weakly changed; i.e., they seem to be weakly effective for revealing the changes induced by VP in higher plants.

## 5. Materials and Methods

We used the spectra of reflected light and values of photosynthetic parameters (Y(II) and NPQ), which were measured in our earlier work [65]. Details of the experimental procedure were described in the work [65].

Briefly, pea seedlings (*Pisum sativum* L., 14–21 days old) were hydroponically cultivated in a Binder KBW 240 plant growth chamber (Binder GmbH, Tuttlingen, Germany).

VP was induced by burning the leaflet in the first leaf of the pea seedling (flame, 3–4 s, approximately 1 cm^2^). The leaflet was burned after 75 min of adaptation of the seedling in the system for measurements of photosynthetic and reflectance parameters.

Photosynthetic and reflectance parameters, which were measured in the second leaves of the pea seedlings, were used in the analysis.

A Pulse-Amplitude-Modulation (PAM) fluorometer Dual-PAM-100 (Heinz Walz GmbH, Effeltrich, Germany) was used for photosynthetic measurements (Y(II) and NPQ). The photosynthetic measurements were initiated after 15 min dark adaptation before turning on the actinic light.

A compact wide-range spectrometer S100 (SOLAR Laser Systems, Minsk, Belarus) and a fiber optics cable were used for measurement of reflected light. The measurements of the reflected light were initiated after 30 min illumination by the actinic light (30 min before VP induction). A white card (QPcard 101 Calibration Card v3, Argraph Corp., Carlstadt, NJ, USA) was used as the reflectance standard for calibration of the reflected light.

A halogen lamp (Osram Decostar, 3000 K, 20 W, 12 V, Germany) was used as the source of the white actinic light. The distance from the lamp to the investigated leaf was approximately 15 cm; the intensity of the leaf illumination by the actinic light was approximately 630 µmol m^−2^ s^−1^. The duration of illumination before VP induction was 60 min.

## Data Availability

The data presented in this study are available upon request from the corresponding author.
